# Flexibility of foraging strategies of the great skua *Stercorarius skua* breeding in the largest colony in the Barents Sea region

**DOI:** 10.1186/s12983-018-0257-x

**Published:** 2018-03-23

**Authors:** Dariusz Jakubas, Lech M. Iliszko, Hallvard Strøm, Halfdan H. Helgason, Lech Stempniewicz

**Affiliations:** 1 Department of Vertebrate Ecology and Zoology, Faculty of Biology, University of Gdańsk, Wita Stwosza 59, 80-308 Gdańsk, PL Poland; 2 Norwegian Polar Institute, Fram Centre, Postboks 6606, Langnes, 9296 Tromsø, Norway

**Keywords:** Seabird, Arctic, Foraging behaviour, Individual foraging specialization

## Abstract

**Background:**

Foraging strategies of seabird species often vary considerably between and within individuals. This variability is influenced by a multitude of factors including age, sex, stage of annual life cycle, reproductive status, individual specialization and environmental conditions.

**Results:**

Using GPS-loggers, we investigated factors affecting foraging flight characteristics (total duration, maximal range, total distance covered) of great skuas *Stercorarius skua* of known sex breeding on Bjørnøya, Svalbard, the largest colony in the Barents Sea region. We examined influence of sex (females are larger than males), phase of breeding (incubation, chick-rearing), reproductive status (breeders, failed breeders) and bird ID (they are known for individual foraging specialization). Our analyses revealed that only bird ID affected foraging flight characteristics significantly, indicating a high degree of plasticity regardless of sex, reproductive status or phase of breeding. We recognized three main groups of individuals: 1) those preying mainly on other seabirds in the breeding colonies (6%), 2) those foraging at sea (76%) and kleptoparasiting other seabirds and/or foraging on fish and/or offal discarded by fishing vessels, and 3) those alternating between preying on other seabirds in breeding colonies and foraging at sea (18%). Despite marked size sexual dimorphism, we found no apparent sex differences in flight characteristics. Birds after egg- or chick-loss and thus not constrained as central foragers did not modify their foraging flights.

**Conclusions:**

Great skuas breeding on Bjørnøya displayed a high degree of plasticity regardless of sex, reproductive status or phase of breeding. We recognized groups of individuals regularly preying in the seabird colonies, foraging at sea, and alternating between both strategies. This suggests foraging specialization of some individuals.

## Background

Foraging strategies of seabird species often vary considerably between and within individuals. This variability results from targeting usually widely-dispersed, spatiotemporally patchy prey and the influence of a multitude of other factors including age, sex, stage of annual life cycle, reproductive status, individual specialization and environmental conditions. These factors have major implications for our understanding of seabird ecology, because they affect the use of resources, level of intra-specific competition and niche partitioning [1].

Sexual differences in foraging behaviour are apparent in many seabirds. This may reflect habitat specialization and/or sex-specific nutrient requirements [2–4] and reduces competition between males and females. Sexual size dimorphism is thought to play a functional role in flight performance and is used to explain differences in the at-sea distribution of male and female seabirds [5]. Partial or complete sexual foraging segregation has been reported in several seabird groups including both sexually dimorphic and monomorphic species (e.g. [6, 7]). Sexual segregation in foraging may be especially expressed in years of greater environmental stochasticity, when food availability is reduced [8].

Age may affect foraging strategy considerably, and immature birds avoid competition with adults by feeding in suboptimal habitats which increases their foraging time (e.g. [9–11]). Younger or less experienced breeders may forage less efficiently and/or at a lower trophic level, which may be reflected in reduced breeding performance compared to older birds [9, 12–14]. Immatures may be far more exploratory and lack route or foraging site fidelity compared to adult breeders [11]. Inferior foraging success in younger individuals reflects their poorer skills in identifying, catching or handling prey or in selecting suitable locations, and weaker motor control or physiological fitness [1].

Within a single breeding season, seabirds may adopt a temporally flexible foraging strategy to satisfy different energy and time demands of incubation and chick-rearing and both their own energetic requirements and those of their offspring. The changing degree of central-place constraint in successive phases of the breeding and seasonal changes in prey availability may affect distribution, activity patterns or diet of seabirds [4, 15, 16]. Foraging strategies may also vary within a single breeding season as a response to fluctuations in prey availability that is driven by prey biology, environmental conditions and/or prey depletion near the colony due to intense foraging (“Storer-Ashmole’s halo” [17–20]). Moreover, some species (albatrosses, petrels, little auk *Alle alle*) adopt an unimodal foraging strategy during incubation (trip of similar duration) and bimodal strategy during chick-rearing, alternating short trips to nearby locations to collect food for chicks, and long trips to further location mainly for self-feeding (e.g. [21–24]).

One of the less studied factors affecting foraging strategy is reproductive status. Failed breeders often continue to associate with the colony, operating as central-place foragers but expand their foraging areas [25]. They may be partially or completely segregated from breeders, probably to avoid competition [1].

Many marine birds and mammals show individual feeding specializations in terms of distribution, behaviour, diet or other aspects of resource acquisition that remain after accounting for the group effects of sex, age, breeding stage and status [26, 27]. Individual specialization may be expressed in fidelity to feeding sites, consistency in foraging trip characteristics, diving patterns and other at-sea activity, habitat use, specific prey items or trophic level in the short or long term [1]. Foraging specializations are probably learned during individual exploratory behaviours early in life, which then become canalized with age and experience [28, 29].

In this study, we investigated foraging flights of great skuas *Stercorarius skua* breeding on Bjørnøya (Svalbard), one of the northernmost breeding areas within this species’ range [30]. The great skua is a dietary generalist exploiting a wide range of prey. It searches for and catches food exclusively on the wing, mainly by splash-diving onto surface fish shoals. It may also kleptoparasitise and prey on seabirds or forage on discards from fishing vessels [31, 32]. The great skua is an important predator of seabirds during the breeding season taking eggs, chicks and adult birds. Dietary specialization of great skua pairs and colonies has been documented in several studies [5, 33–36]. Remains of seabirds (mainly black-legged kittiwakes *Rissa tridactyla* and northern fulmars *Fulmarus glacialis*) and fish were found in 98 and 38% of pellets of great skuas breeding on Bjørnøya in 2008–2009. Considerable fractions (33 and 61% in 2008 and 2009, respectively) of pairs breeding there showed dietary specialization on seabirds (≥70% pellets contained only seabird remains) [37]. The great skua breeds mainly colonially on flat or gently sloping ground. The female lays 1–2 eggs that are incubated for 26–32 days by both sexes, although mainly by the female. The semi-altricial chicks stay in the nest area for 40–51 days after hatching. The chicks are guarded mainly by the female and fed by both parents [30]. The great skua and other skuas (Stercorariinae), like other birds with a raptorial lifestyle, display sexual size dimorphism with females being larger than males [5].

Despite extensive studies on the great skua foraging and breeding ecology (e.g. [35, 38, 39]), foraging flights during the chick-rearing period have been studied so far only in Shetland [35] and St. Kilda, Outer Hebrides [40].

The aim of this study was to investigate factors affecting foraging flight characteristics (maximal range, total distance covered and total duration) of great skuas breeding on Bjørnøya. We considered sex, stage of breeding (incubation or chick-rearing), reproductive status (breeders vs. failed breeders) and bird ID. We expected that:

1. Because of various time budgets of incubating and chick-rearing individuals, birds during incubation would have longer foraging flights than during chick-rearing given the necessity of adults to feed chicks in regular intervals;
2. Due to various time budgets and duties, failed breeders free of central place forager constraint would have longer foraging flights to more distant areas enabling them to feed beyond the cost-effective flight distance to the colony;
3. Regardless of breeding status and phase of the breeding period and considering sexual size dimorphism, larger females will perform flights of longer duration and range compared to smaller males;
4. Individual great skuas that specialized in foraging on local resources (other seabirds on the same island) would perform shorter trips characterized by high repeatability in utilisation densities.

## Methods

### Study area

**Fig. 1:**
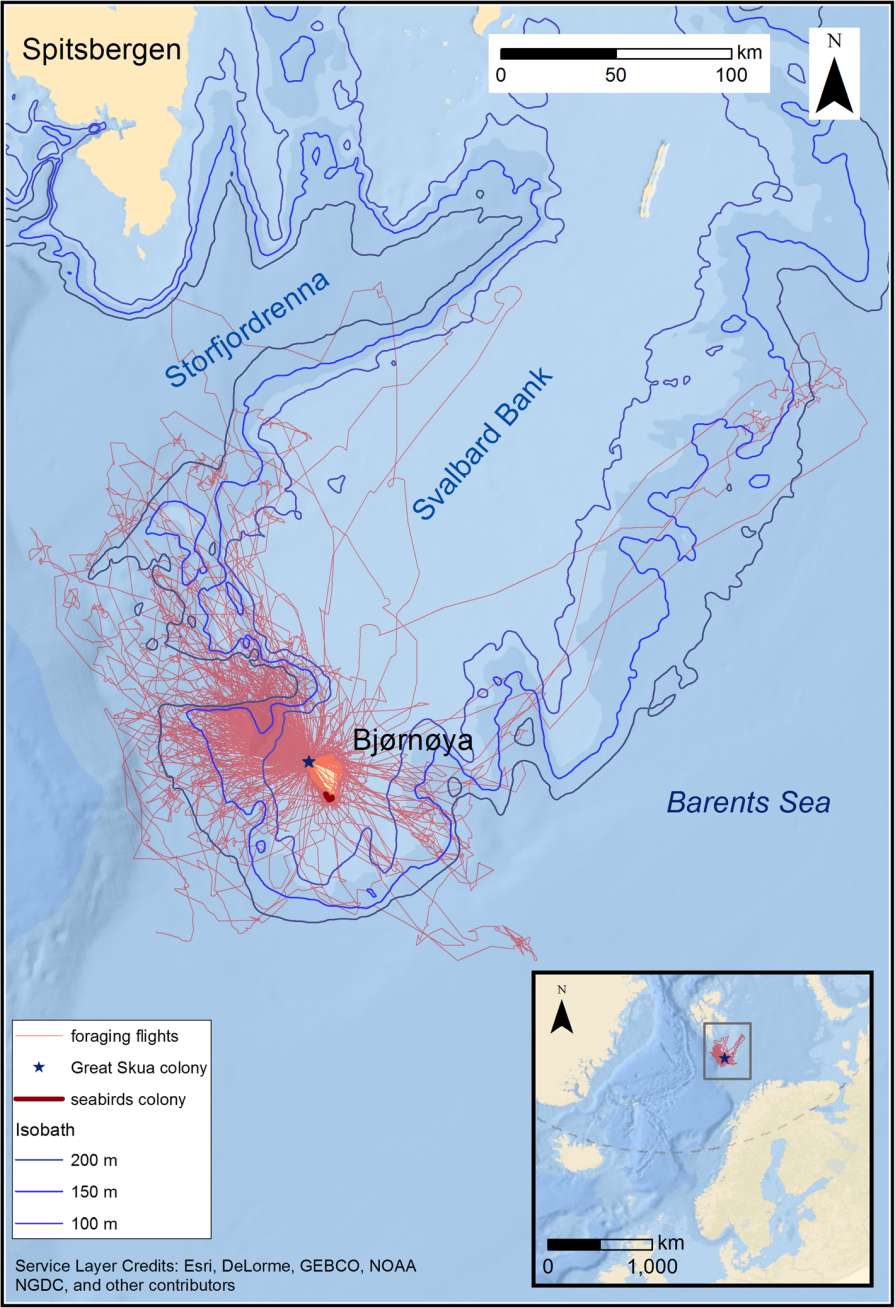
All foraging flights of GPS-tracked great skuas breeding on Bjørnøya. Blue star indicates location of the great skua breeding colony, red narrow lines foraging flights, red thick line location of the seabird (kittiwakes and guillemots) colonies. Isobaths 100–250 (blue lines) define the position of Polar Front separating cold Artic and warm Atlantic water masses [41, 42]

Bjørnøya (74°30′N, 19°01′E) is a 178 km^2^ island in the western Barents Sea at the south-western edge of the shallow Spitsbergen Bank (Fig. 1). This bank is, in summer, characterized by a mixture of cold Arctic water and melt-water from the Barents Sea ice. The waters around Bjørnøya are characterized by non-stratified Spitsbergen Bank water near the island. This water is surrounded to the south and west by a frontal zone where cold fresher Arctic water mixes with warm saline Atlantic water that is found in the deeper parts to the west, south and southeast of Bjørnøya. The zone where the two water masses meet is called the Polar Front. Its location is determined mainly by bathymetry and is oriented parallel to the slopes of the shallow Spitsbergen Bank with the surface expression following the 100–250 m isobath [41–43]. Bjørnøya accommodates large colonies of seabirds including black-legged kittiwakes, Brünnich’s guillemots *Uria lomvia*, common guillemots *U. aalge* and little auks [44]. Great skuas were first observed breeding on Bjørnøya in 1970 [30, 44] but the population size has since increased rapidly, becoming the largest colony in the Barents Sea region [currently estimated at 750–1000 breeding pairs (H. Strøm unpubl. data) and one of the northernmost within the species’ range.

### GPS tracking

To characterize foraging flights of great skuas, we used global positioning system (GPS) loggers (Harrier, Skua and Uria models, Ecotone, Sopot, Poland) recording time, position and momentary speed. We deployed 21 loggers on one pair member from 21 nests. We captured the birds on nests during the incubation period in June 2014 in the colony in Flakmyrvatna area in NW part of the island. The study area contained 107 breeding pairs. The logger weight (including attachment, Harrier: 19.1–19.3 g; Skua: 26.3 g; Uria: 11.2 g) was equivalent to 1.2–1.5% (Harrier, *N* = 14 individuals), 1.7–2.1% (Skua, *N* = 4) and 0.7–0.9% (Uria, *N* = 3) of the bird’s body mass. The lightest logger type (Uria) was attached to the tail feathers. Other types of loggers were attached with full harness (12 Harriers, 4 Skuas) or with a loop around head and tail (2 Harriers). The GPS-loggers used a bidirectional radio link with base stations installed in the colony, allowing remote data download. To save battery life, the base station automatically switched off the loggers while they were within the download range of the base station. The loggers restarted to record positions when birds left the base station signal range. Sampling interval was set to 15 min; however, when the battery voltage was low, the interval increased to 60 min. The field-tested accuracy of the GPS receiver was ±10 m for 95% of positions. We analysed only records with sampling rates of up to 30 min. Within longer intervals between recorded positions, birds may have had time to return to the colony and start a new trip.

We sexed the studied birds molecularly based on DNA extracted from blood following a modified protocol of [45]. We amplified CHD genes from extracted DNA by PCR using primers 2550F and 2718R [46]. Analyses were carried out at the Norwegian University of Science and Technology, Trondheim.

We received data from 17 of 21 individuals. In the case of three loggers, communication with the base station failed and in the last case, the logger changed recording intervals, forcing us to exclude data from the analyses. In total, 402 trips of 17 birds were analysed with 4 to 87 trips recorded per individual (Table 1).

**Table 1:** Trips characteristics of GPS-equipped great skuas breeding on Bjørnøya

		No of flights of GPS-tracked birds				
Bird ID	Sex	BEL	AEL	BCD	ACD	Total
H17	F	13	18	–	–	31
H18	F	15	–	–	–	15
H19	F	3	5	–	–	8
H20	F	14	–	–	–	14
H21	F	23	–	2	21	46
H22	F	22	–	–	–	22
H24	F	13	21	–	–	34
H25	F	2	2	–	–	4
H26	M	5	70	–	–	75
H27	M	5	–	–	–	5
H28	F	7	2	–	–	9
H29	M	4	8	–	–	12
H30	F	7	–	–	–	7
S07	M	27	–	36	24	87
S08	F	1	8	–	–	9
S09	M	11	2	–	–	13
U14	M	11	–	–	–	11
Total	_	183	136	38	45	402
No of birds	6 M 11F	17	9	2	2	17

Number of flights at particular phases: *BEL* incubation before egg loss, *AEL* incubation after egg loss, *BCD* chick rearing before chick loss, *ACD* chick rearing after chick loss

To monitor the status of GPS-logger equipped individuals and their nests, we controlled the nest content 4–10 d days after the logger deployment. We found that all studied great skuas failed breeding. Among the 21 nests, 53% failed at the incubation stage. In the remaining 47%, at least one chick hatched but all died after 1 to 17 days. Similar breeding failure was recorded in the whole colony area, where no birds were caught. No chicks reached the age of 20 days in 107 nests monitored. We do not know the precise reason of this failure, but we observed frequent conspecific predation which can be related to unfavourable feeding conditions (HS – unpubl. data).

### Statistical analyses

Based on the geographical positions recorded by the GPS-loggers, we analysed the following foraging flights characteristics: (1) maximum range of flights – distance from the colony to the distal point reached on each foraging trip; (2) the total distance covered (km) as the sum of the distances (km) between all GPS positions along each individual’s track; in the case of incomplete trips (i.e. without the first or last position), the missing part was estimated based on distance of the first/the last position nearest to the colony; (3) total trip duration, defined as the time between departure and return to colony; in the case of the incomplete trips, the lacking part was estimated based on the momentary speed and distance of the first/the last position nearest to the colony. To characterize the whole area utilized by studied individuals, we calculated minimal convex polygon (MCP).

Considering the sample size for particular studied features (sex, reproductive status, phase of breeding), we were able to perform analyses including sex and ID of birds for the incubation periods for 17 individuals. For nine individuals, we compared flight characteristics during incubation before and after eggs loss. For one male and one female, we were able to compare flight characteristics during incubation, chick-rearing and after chick loss.

To characterize flights of males and females during the incubation period, we used Conditional Inference Tree (CIT). This is a non-parametric class of regression tree, examining the relationship between multiple explanatory variables and a single response variable using a recursive binary-partitioning process. Model outputs produce an ‘inverted tree’, in which the root at the top contains all observations, which is divided into two branches at the node. The aim of splitting the data at each step is to establish groups that had a between-variation as large, and within-variations as small, as possible. The node provides information about the explanatory variable name and its probability value. Branches are further split into two subsequent nodes and so on. [47]. CIT uses a machine learning algorithm to determine when splitting is no longer valid using statistically-determined stopping criterion, an a priori *p* value [48]. CIT is robust to typical regression problems such as over-fitting, collinearity, and bias with regard to the types of explanatory variables used [47, 48]. We conducted CIT analyses in R software [49] using *party* package [48]. We checked significance of nodes in CIT analyses using structural change test implemented in *strucchange* package in R [50].

For data from the incubation period with satisfactory sample size, we calculated utilization distributions (UD, a probability distribution constructed from data providing the location of an individual in space at different points in time) for home ranges (95% kernel density) and core ranges (50% kernel density) of males and females in Geospatial Modelling Environment (GME) ver. 0.7.4.0 software (www.spatialecology.com/gme/) using *kde* function using plugin bandwidth selection. To investigate overlap between utilization distributions (i.e. 95 and 50% kernel density isopleth) of males and females during the incubation period before egg loss, individuals before and after egg loss, and individuals during incubation and chick-rearing periods, we used *adehabitat* package in R [51] with algorithm BA, i.e. the Bhattacharyya’s affinity, a statistical measure of affinity between two populations. It ranges from zero (no overlap) to 1 (identical UDs) [52]. To measure individual consistency in UD between particular trips, we calculated the mean Bhattacharyya’s affinity of all pairwise combinations of the recorded trips and then calculated the mean value for particular individuals.

To estimate how many flights were to seabird colonies at the south end of Bjørnøya, we counted all flights within a 1 km buffer around the seabird cliff. We mapped data from the GPS loggers, performed spatial analyses and produced all figures with maps using ArcMap/ArcGIS 10.3.1 (Environmental Systems Research Institute, Redlands, CA, USA).

## Results

Great skuas breeding on Bjørnøya performed foraging flights lasting 0.8–224 h (median 5.2 h, *N* = 17 individuals), reaching areas at maximal distance from the colony ranging from 1.6 to 1003 km (median 88.1 km), covering total distances ranging from 3.6 to 2790 km (median 199 km) (Table 2, Fig. 1). Minimal convex polygon of all GPS position recorded ranged from 0.03 to 16,946 km^2^ (median 141.7 km^2^) (Table 2).

**Table 2:** Trips characteristics of GPS-equipped great skuas breeding on Bjørnøya

Bird	Total flight duration [h]			Maximal range [km]			Total distance covered [km]			MCP[km^2^]			Mean BA	
ID	Med	Q1	Q3	Med	Q1	Q3	Med	Q1	Q3	Med	Q1	Q3	HR	CR
H17F	9.6	4.2	21.8	89.6	45.6	236.5	207.0	118.4	599.0	289.9	124.3	1621.8	0.36	0.08
H18F	4.7	4.2	6.8	120.1	81.9	146.1	247.4	164.4	295.0	156.8	66.8	275.9	0.68	0.23
H19F	9.4	4.7	21.9	124.0	94.8	146.9	266.3	205.0	531.1	232.0	113.3	1035.3	0.54	0.16
H20F	3.7	2.6	6.6	76.2	47.6	163.0	154.0	116.6	329.9	100.8	27.0	325.9	0.48	0.16
H21F	5.5	3.7	9.4	127.5	86.6	191.7	279.5	148.1	416.3	266.9	98.0	776.9	0.49	0.08
H22F	4.7	3.1	7.4	80.4	63.9	176.3	222.1	135.3	354.4	153.1	42.3	313.8	0.61	0.16
H24F	4.2	3.1	5.2	90.1	65.5	117.2	201.8	134.4	262.1	144.4	42.6	337.0	0.69	0.20
H25F	61.0	27.1	118.4	263.8	128.8	657.9	1082.2	491.3	2011.0	4629.7	1770.0	10,438.4	0.42	0.17
H26M	6.0	4.2	8.9	41.4	35.7	51.7	105.1	82.9	147.9	45.4	24.9	104.5	0.50	0.17
H27M	17.8	14.6	18.5	296.1	222.6	315.0	651.1	540.6	681.8	1439.7	1432.5	1718.0	0.68	0.19
H28F	11.0	4.5	11.9	114.9	65.7	126.8	259.0	236.2	312.3	282.6	104.1	525.9	0.41	0.11
H29M	14.1	3.0	22.1	150.5	83.6	290.0	336.5	170.9	715.3	788.5	70.7	2412.6	0.44	0.06
H30F	2.1	1.9	3.7	31.7	29.1	34.4	74.7	68.0	88.0	29.4	23.5	39.2	0.79	0.32
S07 M	4.1	3.0	5.6	99.3	60.9	129.1	209.9	129.9	301.8	129.4	45.7	341.1	0.62	0.21
S08F	6.0	3.0	35.6	105.6	62.8	269.2	273.0	126.4	905.0	509.7	132.9	3799.6	0.45	0.13
S09 M	11.5	9.5	24.0	168.3	125.9	247.3	439.4	318.5	841.8	1053.9	383.6	2733.7	0.55	0.10
U14 M	6.2	3.7	10.8	65.0	47.8	90.2	189.2	146.4	219.5	132.3	58.5	476.2	0.64	0.17
All	5.2	3.5	9.4	88.1	45.8	146.1	199.0	117.3	336.0	141.7	42.9	496.8	0.54	0.19

*MCP* minimal convex polygon, *Bird ID* bird ID with coded sex (F - female, M – male), *N* number of trips, *Med* median, *Q*_*1*_*, Q*_*3*_ percentiles 25 and 75%, *Mean BA* mean values of the Bhattacharyya’s affinity, *HR* home range (utilisation density 95%), *CR* core range (utilisation density 50%)

### Factors affecting foraging flights characteristics during incubation

**Fig. 2:**
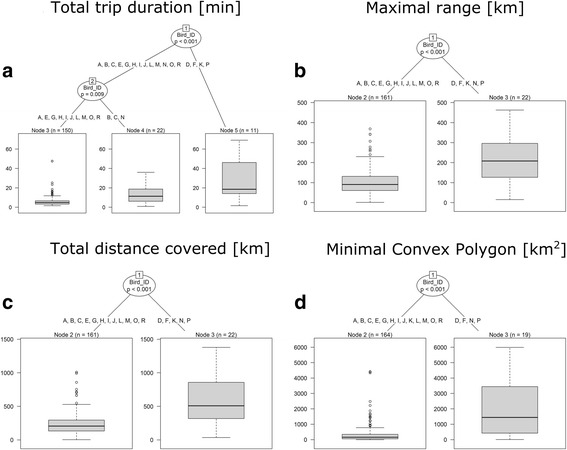
A Conditional Inference Tree characterizing flights of GPS-logger-equipped great skuas incubating eggs on Bjørnøya. **a** Total trip duration. **b**. Maximal ranges of flights. **c** Total distance covered. **d** Minimal convex polygon. The following characteristics of recorded great skua flights: sex (F – female, M - male), Bird_ID (individuals A-P) were used as initial explanatory variables. Encircled variables have the strongest association to the response variable (maximal ranges of flights). The *p* values listed at each encircled node represent the test of independence between the listed variable (sex, Bird_ID) and the response variable (maximal ranges of flights). Terminal nodes indicate which variable levels characterizing the great skua flights ranges and *n* indicates the number of flights corresponding to specific Bird_ID levels. Boxplots show the median (band inside the box), the first (25%) and third (75%) quartile (box), the lowest and the highest values within 1.5 interquartile range (whiskers) and outliers (circles)

To investigate factors affecting foraging flight characteristics during incubation (we considered only the period before egg loss), we used bird ID and sex as explanatory variables. The Conditional Inference Tree (CIT) results indicate that bird ID best characterized total foraging trip duration variability. CIT recognized three groups of individuals with various mean foraging times: 29.2 h (node 5 represented by 4 individuals), 13.8 h (node 4 represented by 3 individuals) and 6.3 h (node 3 represented by 10 individuals) (Fig. 2a).

In the case of maximal range of foraging flights, bird ID characterized the best observed variability. Two main nodes were recognized: 106.6 km (node 2 represented by 12 individuals) and 212.4 km (node 3 represented by 5 individuals) (Fig. 2b).

CIT results indicated that bird ID best characterized total distance covered. Two main nodes: 246.8 km (node 2 represented by 12 individuals) and 613.8 km (node 3 represented by 5 individuals) (Fig. 2c) were recognized.

In the case of maximal convex polygon of all recorded GPS positions, bird ID characterized the best observed variability with two main nodes: 81.4 km^2^ (node 2 represented by 13 individuals) and 2085.1 km^2^ (node 3 represented by 4 individuals) (Fig. 2d).

**Fig. 3:**
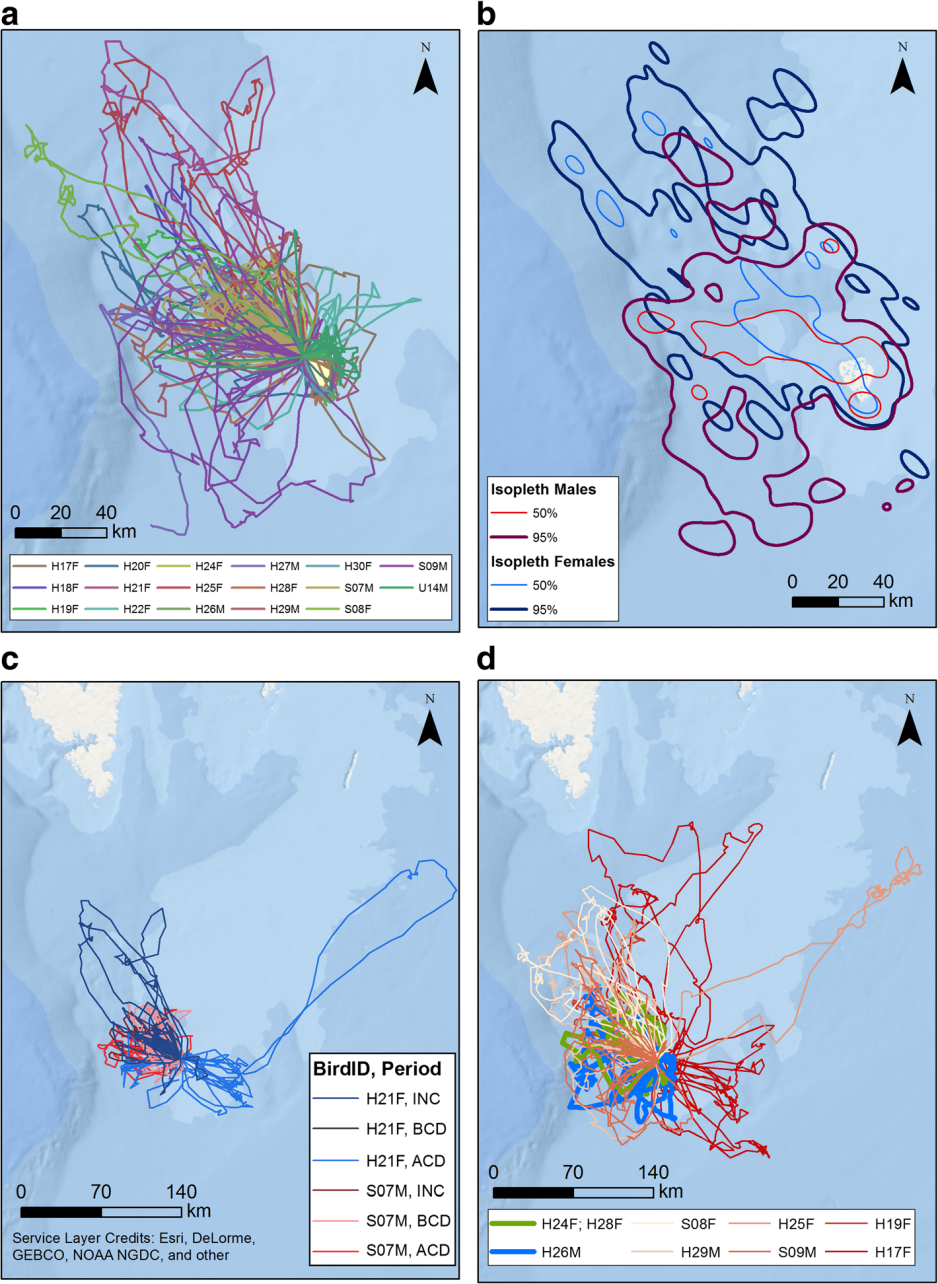
Characteristics of foraging flights of great skuas breeding on Bjørnøya. **a** Flights of all individuals during incubation excluding period after egg loss. **b** 95% kernel home ranges and 50% core ranges of great skua females (blue) and males (red) during incubation excluding period after egg loss. **c** A comparison of flights of two individuals (male red, female blue) during incubation (INC) and chick rearing period, before (BCD) and after chick loss (ACD). **d** A comparison of flights of nine individuals during the incubation period including period after egg loss; group of flights characterized by sex differences indicated in green (females) and blue (male); flights of other individuals indicated in different colours (D)

In the case of individuals incubating eggs (11 females and 6 males), the 95% utilisation distributions showed Bhattacharyya’s affinity (BA) 0.80 between females and males (Fig. 3). In the case of core range, BA values were lower: 0.42 (Fig. 3b).

### Factors affecting foraging flights of birds that lost eggs

**Fig. 4:**
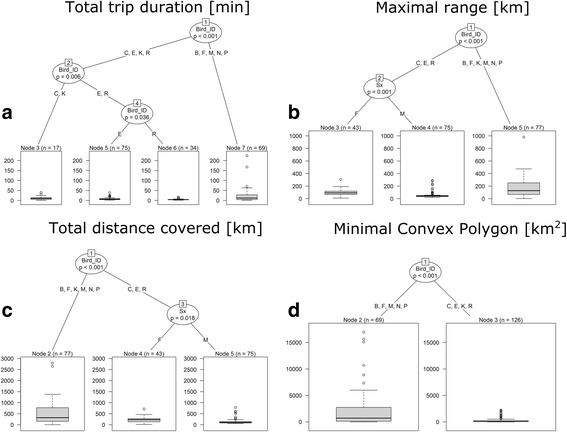
A Conditional Inference Tree characterizing flights of GPS-logger-equipped great skuas breeding on Bjørnøya during the incubation period (including periods before and after egg loss). **a** Total trip duration. **b** Maximal ranges of flights. **c** Total distance covered. **d** Minimal convex polygon. The following characteristics of recorded great skua flights: sex (Sx; F – female, M - male), status (BEL – before egg loss, AEL – after egg loss), Bird_ID (individuals A-P) were used as initial explanatory variables. Encircled variables have the strongest association to the response variable (maximal ranges of flights). The *p* values listed at each encircled node represent the test of independence between the listed variable (sex, Bird_ID) and the response variable (maximal ranges of flights). Terminal nodes indicate which variable levels characterizing the great skua flights ranges and *n* indicates the number of flights corresponding to specific sex or Bird_ID levels. Boxplots show the median (band inside the box), the first (25%) and third (75%) quartile (box), the lowest and the highest values within 1.5 interquartile range (whiskers) and outliers (circles)

To characterise foraging flight of great skuas during the incubation period (including periods before and after egg loss), we used sex, status (BEL – before egg loss, AEL – after egg loss), and bird ID as explanatory variables. CIT results indicated that bird ID and sex best characterized total foraging trip duration variability. CIT recognized two main groups – node 2 represented by 5 individuals with mean total trip time 22.8 h, and node 3. The latter node was split into two nodes: node 7 (represented by 2 individuals and mean trip duration 12.1 h) and node 4. The latter node was split between sexes. In that group containing 3 individuals, females performed flights of shorter duration (mean 5.0 h) than the male (8.0 h) (Fig. 4a).

In the case of maximal range of foraging flights, bird ID and sex characterized the best observed variability. Two main nodes were recognized: node 2 represented by 6 individuals and characterized by more distant flights (mean 174.3 km) and node 3. The latter node represented by 3 individuals was split by sex. Females were characterised by higher maximal range of foraging flights (mean 96.4 km) compared to the male (58.4 km) (Fig. 3c, 4b).

In the case of CIT total distance covered during foraging flights, bird ID and sex characterized the best observed variability. Two main nodes were recognized: 518.8 km (node 2 represented by 6 individuals) and node 3 represented by 3 individuals. The latter node was split by sex. Females were characterised by higher total distance covered during foraging flights (mean 230.8 km) compared to the male (154.1 km) (Fig. 4c).

In the case of maximal convex polygon of all recorded GPS positions, bird ID characterized the best observed variability. Two main nodes were recognized: 2207.7 km^2^ (node 2 represented by 5 individuals) and 262.9 km^2^ (node 3 represented by 4 individuals) (Fig. 4d).

In the case of nine incubating individuals that lost eggs, the 95% utilisation distributions showed a Bhattacharyya’s affinity (BA) of 0.74 between the phases before and after egg loss. In the case of core range, the BA value was lower - 0.36.

### Factors affecting foraging fights of individuals that lost chicks

**Fig. 5:**
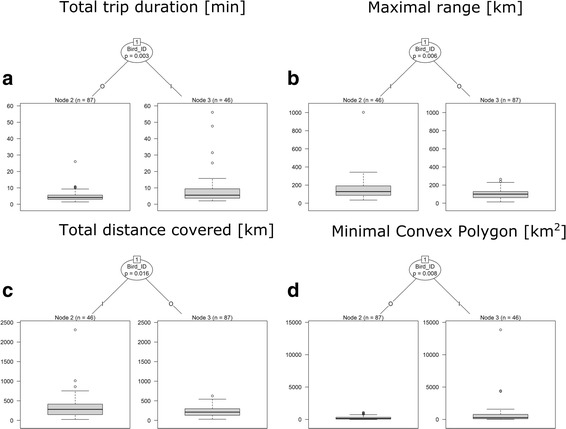
A Conditional Inference Tree characterizing flights of two GPS-logger-equipped great skuas breeding on Bjørnøya during the incubation and chick-rearing periods (including periods before and after chick death). **a** Total trip duration. **b** Maximal ranges of flights. **c** Total distance covered. **d** Minimal convex polygon. The following characteristics of recorded great skua flights: status (INC – incubation, BCD – chick rearing before chick death, ACD - after chick death), Bird_ID (I – female, O – male) were used as initial explanatory variables. Encircled variables have the strongest association to the response variable (maximal ranges of flights). The *p* values listed at each encircled node represent the test of independence between the listed variable (status, Bird_ID) and the response variable (maximal ranges of flights). Terminal nodes indicate which variable levels characterizing the great skua flights ranges and *n* indicates the number of flights corresponding to specific Bird_ID level. Boxplots show the median (band inside the box), the first (25%) and third (75%) quartile (box), the lowest and the highest values within 1.5 interquartile range (whiskers) and outliers (circles)

To characterise foraging flights of great skuas during the incubation and chick-rearing periods (including periods before and after chick death), we used status (INC – incubation, BCD – before chick death, ACD – period after chick death), and bird ID (two individuals: I – female and O - male) as explanatory variables. CIT results indicated that bird ID best characterized all characteristics of the flights (Fig. 3c, 5).

We found that in the case of two individuals tracked throughout incubation (INC) and chick-rearing (BCD – before chick death, ACD - after chick death), the 95% utilisation distributions showed Bhattacharyya’s affinities (BA) of 0.71 between INC and BCD, 0.56 between INC and ACD, and 0.36 between BCD and ACD. In the case of core range, the BA values were 0.30 between INC and BCD, 0.25 between INC and ACD, and 0.14 between BCD and ACD.

### Foraging specialisation

**Fig. 6:**
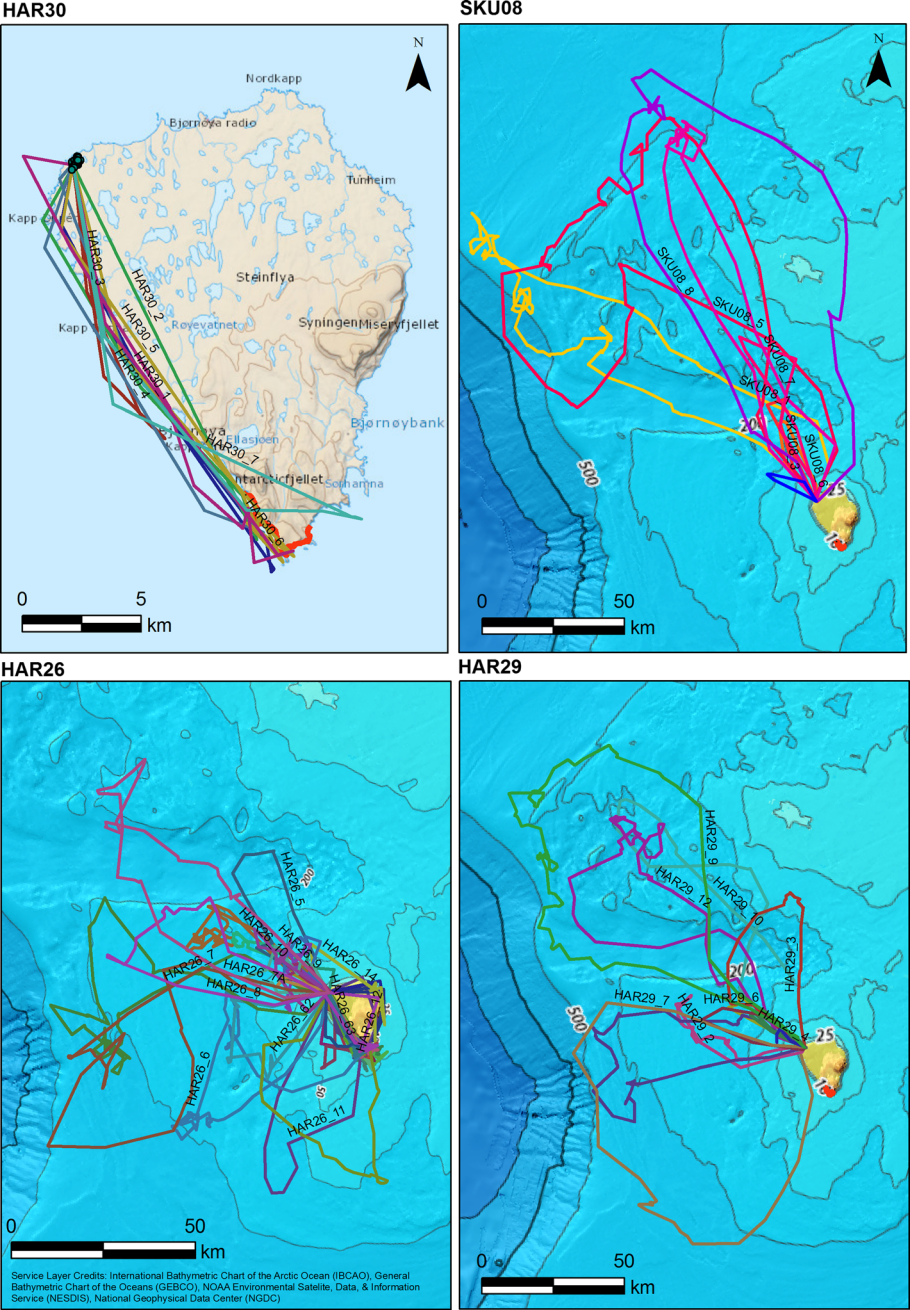
Flights of four GPS-logger-equipped great skuas breeding on Bjørnøya. **HAR30 -** A seabird colonies specialist. **SKU8 and HAR 29 -** Individuals foraging over the shelf zone, **HAR26** - An individual alternating foraging in seabirds colony and over the shelf zone. Colours indicate particular flights of the same individual. Red thick line indicates location of the seabird (kittiwakes and guillemots) colonies in the southern part of the island. Background: bathymetry map (Arctic Ocean Base, https://services.arcgisonline.com/arcgis/rest/services/Polar/Arctic_Ocean_Base/MapServer)

One individual, the female HAR 30, tracked throughout incubation, flew exclusively towards the seabird colonies at the south end of the island (Fig. 6a). The majority of her flights (6 of 7; 86%) targeted the seabird colonies (i.e. within the 1 km buffer zone around the colony). Her flights were characterized by the shortest total duration time, the lowest range, total distance covered and MCP area of all the studied individuals (Table 2). Three other individuals (females HAR 17, HAR 20 and male HAR 26) of 17 studied (17.6%), regularly visited the same colonies, but also flew to other areas. Flights within the 1 km buffer around the colony made up 13%, 29% and 11% of all recorded flights of HAR17, HAR20 and HAR26, respectively. Remaining individuals (76%) flew out to sea, mainly in a NW direction from the colony (Fig. 6).

The consistency of utilisation density of particular flights (i.e. mean BA values per individual) ranged from 0.36 to 0.79 in the case of home range (HR, 95% kernel density) and from 0.06 to 0.32 in the case of core range (CR, 50% kernel density) (Table 1). The trips of HAR 30 were characterized by the highest consistency (the highest values of mean Bhattacharyya’s affinity) of both HR and CR. Almost half (47%) of the studied great skuas had mean BA values for HR higher than average value for all individuals. In the case of CR, only 29% individuals were characterized by BA values higher than average value.

## Discussion

Our study revealed that individual specialization had the greatest effect on foraging flights of great skuas breeding on Bjørnøya. Three main strategies were identified: foraging on other seabirds, foraging at sea and a generalist strategy mixing the two. Similarly, great skuas from Unst, Shetland exhibited dietary specialization: a small proportion fed almost exclusively upon seabirds, a small proportion fed as generalists and most feed on fishery discards [35]. Also the southern hemisphere species, the brown skua *Stercorarius antarcticus lonnbergi* breeding on King George Island (Maritime Antarctica) adopted three main strategies; foraging in a penguin colony, in a storm-petrel colony and at sea [53]. Individual specialization may have a selective advantage where resources are to some extent predictable. The specialist foraging preference is probably passed to offspring through learning as they need to develop particular skills to successfully pursue different foraging modes including kleptoparasitism, predation of selected prey species or scavenging [1, 35, 54].

The study from Unst, Shetland revealed that great skuas specialized in seabird predation were able to spend less time foraging than individuals feeding predominantly on fish in the open sea [35]. In concordance with this, the total trip duration of the female from Bjørnøya that specialized in foraging in the seabird colonies (median 2.1 h) was similar to the mean values 1.1–3.0 h reported for the radio-tracked seabird specialists from Shetland [35]. The proportion of great skuas foraging mostly in seabird colonies in our study (5.8%) was lower than the frequency of seabird specialists calculated based on pellet composition analyses performed in the same colony, i.e. 33% in 2008 and 62% in 2009 [37]. It is, however, possible that great skuas also killed seabirds at sea, not only in the colonies.

Great skuas breeding on Bjørnøya flew further (up to 1003 km) than birds from Foula, Shetland (up to 219 km) [55]. On Foula, the frequency of nearby foraging flights (up to 40 km) was higher (~ 40%) compared to Bjørnøya (15%) (Table 3). This may be attributed to a lower proportion of terrestrial foraging areas on Bjørnøya.

**Table 3:** Comparison of frequencies of foraging flight ranges of GPS logger equipped great skuas breeding on Bjørnøya (this study) and on the Foula, Shetland, UK [55]

Breeding site	Distance from colony				
	< 20 km	20–40 km	60–80 km	100–120 km	max
Bjørnøya	2%	13%	15%	9%	1003 km
Shetland	~ 16%^a^	24%	17%	11%	219 km

^a^as determined from Fig. 6 in [55]

The mean foraging trip duration in our study (5.2 h) was similar to 4.95 h reported for great skuas from Unst, Shetland that specialized in feeding on fish [35]. However, the minimum convex polygons of great skuas breeding on Unst (mean values 1.0–14.4 km^2^) were considerably smaller than those in our study (median values 29.4–4629.7 km^2^). This may be explained by the higher proportion of individuals specialized as local seabird predators on Unst (20%) than on Bjørnøya (6%) [35]). Moreover, some pairs of great skuas on Unst defended feeding territories within a section of seabird colony [35] and thereby diminished considerably their minimum convex polygon.

The majority of the individuals studied on Bjørnøya flew out to sea, over the shallow shelf area and along the shelf break slope (Fig. 1). The Svalbard Bank, with a minimum depth of less than 40 m is considered as the most productive area in the Barents Sea with primary production about 2–3 times higher than in the adjacent, deeper waters [56]. The shelf break zone is also a good foraging habitat for zooplanktivorous and piscivorous fish and birds including black-legged kittiwakes, Brünnich’s guillemots, common guillemots and little auks [43, 57–59]. It is thus possible that the studied great skuas may kleptoparasite or/and prey on seabirds foraging there or returning to the colony. A great skua dietary study from Bjørnøya from 2008 to 2009 revealed that most of fish otoliths found in pellets originated from Gadiformes fish [37]. Most of the species within this group are mid-water or bottom dwelling species [59] and thus probably only available as discards from fishing vessels [60]. This source of food is a locally important diet component of the great skua breeding in the Outer Hebrides [61] or Shetland [34]. The fishing activity in the Barents Sea in 2014 included areas around Bjørnøya [62], but the level of discards is unknown [63]. It has been reported, however, that discards of Atlantic cod *Gadus morhua* from the Norwegian shrimp fishery in the Barents Sea consists mainly of 1- and 2-year-olds [63] (i.e. with mean body length 10.4 and 18.4 cm, respectively [64]) and thus optimal food for great skuas. Great skuas from Bjørnøya may also prey directly on pelagic fish, such as the capelin *Mallotus villosus* during foraging flights at sea by dipping or surface-seizing. This is supported by an observation of adult and chick regurgitates consisting of capelin remains (H. Strøm unpubl. data), although it is also possible that these remains originated from kleptoparasitism. In Shetland, great skua diet composition varied among the colonies and was dependent on the colony size. The majority of individuals from large colonies fed on fish, including discards, and only a small proportion specialised in killing seabirds [65]. With regard to the size and the rapid growth of the Bjørnøya population, it is likely that they have adapted to feed on widely abundant fish such as capelin or fish discarded from fisheries.

Almost half of the studied birds were consistent in their home range areas (mean values of BA higher than the average) suggesting frequent foraging in the same areas during consecutive flights. This would be expected of individuals repeatedly visiting seabird colonies or those exploring areas characterized by similar oceanographic features as fronts in the shelf break zone providing opportunity to find their own fish or kleptoparasitise other seabirds gathered there to forage. Individuals alternating between flights to sea and to the seabirds colonies had lower repeatability of utilisation densities values than the average for all individuals studied.

That neither sex nor phase of reproduction of the studied birds affected foraging flight characteristics significantly was unexpected. This may be interpreted as high inter-individual variability in foraging being more important than other sources of variability. The only sex difference in total trip duration found was in the small subgroup of birds (three individuals) during incubation but, in this case, sex effect was also a proxy for bird ID. Our result corroborates a study of great skuas and other skuas (Stercorariinae) that found no support for the theory that sexual dimorphism evolved as a result of specialized roles during breeding (smaller males with lower wing loading foraging more efficiently due to greater agility) [5].

Surprisingly, after losing eggs or chicks, individuals freed of the restraint of central-place foraging did not modify their flights characteristic as did failed breeding northern giant petrels *Macronectes halli* and southern giant petrels *M. giganteus* [25]. Using similar home ranges before and after egg loss may indicate foraging site fidelity by the Bjørnøya birds. It suggests that feeding grounds utilized by the studied great skuas were at least good enough for self-maintenance.

We are aware of possible limitations of our study. Firstly, we did not know the age of the studied individuals. It has been reported that time spent foraging to provide food for chicks increases with age of great skua parents breeding in Shetland [66]. Secondly, the 2014 breeding season was a complete failure for great skuas from Bjørnøya [67] with no chicks reaching an age beyond 20 days. Furthermore, there was a 26% reduction in the number of breeding pairs (to 107 nests in the whole monitoring area) compared to 2013 (HS, pers. obs.). We suppose that the breeding failure may have been the result of high conspecific predation rate driven by suboptimal feeding conditions on foraging grounds. In 2014, the stock of the only semi-pelagic or pelagic gadoid fish, the polar cod *Boreogadus saida*, which could have been preyed directly by great skuas or indirectly by kleptoparasiting other seabirds (this fish is frequently preyed by Brünnich’s guillemots and black-legged kittiwakes breeding on or sampled around Bjørnøya [57, 68]), was reduced and its distribution shifted northeastwards, beyond the Barents Sea shelf [62]. The capelin stock at the time of the study was also significantly reduced [62, 69] thereby possibly influencing great skuas directly or/and indirectly. In years when capelin is scarce, the piscivorous guillemots breeding on Bjørnøya may rely more on euphausiids for food [57]. In such years, the kleptoparasitic behaviour of great skuas may be less energetically profitable than in years with a higher contribution of fat-rich fish in their diets. In this context, foraging flights characteristics may also have been different than in other seasons. Finally, logger deployment might be considered as a cause of the breeding failure [70] but this was unlikely due to the overall breeding failure recorded throughout the colony. Moreover, other studies of great skuas fitted with GPS devices revealed that foraging flights characteristics and territory attendance rates were similar to those of control individuals [55].

## Conclusions

The characteristics of great skua foraging flights on Bjørnøya during a poor breeding season were individual rather than a response to sex or breeding status (after or before egg or chick loss). Almost half of the studied birds were consistent in their home range areas during consecutive flights suggesting foraging specialization. Some individuals fed regularly in the seabird colonies at the southern part of the island, some foraged at sea in areas with oceanographic features as fronts providing opportunity to kleptoparasiting or even direct hunting for fish, and some birds used both foraging strategies.

Our study demonstrates the effectiveness of GPS-tracking as a tool for investigating foraging specialization of great skuas. Future studies could usefully apply the same approach, ideally supported by diet composition estimation (e.g. stable isotope ratios, pellet composition), to investigate consistency and flexibility in the foraging strategies.
